# Comparative survival analysis of bladder preservation therapy versus radical cystectomy in muscle‐invasive bladder cancer

**DOI:** 10.1002/cam4.6972

**Published:** 2024-02-06

**Authors:** Nai‐Wen Kang, Yin‐Hsun Feng, Kuei‐Li Lin, Yi‐Chen Chen, Chung‐Han Ho, Ching‐Chieh Yang

**Affiliations:** ^1^ Division of Hematology and Oncology, Department of Internal Medicine Chi Mei Medical Center Tainan Taiwan; ^2^ Department of Radiation Oncology Chi Mei Medical Center Tainan Taiwan; ^3^ Department of Medical Research Chi Mei Medical Center Tainan Taiwan; ^4^ Department of Information Management Southern Taiwan University of Science and Technology Tainan Taiwan; ^5^ Department of Pharmacy Chia‐Nan University of Pharmacy and Science Tainan Taiwan; ^6^ School of Medicine, College of Medicine National Sun Yat‐sen University Kaohsiung Taiwan

**Keywords:** bladder preservation therapy, concurrent chemoradiotherapy, muscle‐invasive bladder cancer, radical cystectomy

## Abstract

**Background:**

Bladder preservation therapy is an alternative to radical cystectomy in patients with muscle‐invasive bladder cancer (MIBC). The purpose of this study is to compare survival outcomes between bladder preservation therapy and radical cystectomy in MIBC patients using an Asian nationwide cancer registry database.

**Methods:**

From the Taiwan Cancer Registry database and the Taiwan National Health Insurance Research Database, we identified bladder cancer patients from 2008 to 2018. The patients with urothelial carcinoma and clinical stage T2‐T4aN0‐1 M0 were included. Propensity score matching by age, gender, clinical stage, cT classification, and Charlson Comorbidity Index score was used between those receiving bladder preservation therapy or radical cystectomy. Overall survival (OS), cancer‐specific survival (CSS), and disease‐free survival (DFS) were compared using the Kaplan–Meier method. Multivariate Cox regression models were used to determine the predictive factors of OS, CSS, and DFS.

**Results:**

Following the propensity score matching, 393 MIBC patients were analyzed, 131 (33.3%) receiving bladder preservation therapy and 262 (66.7%) receiving radical cystectomy. After 5 years of the follow‐up period the overall duration was with a median of 15.6 months. The treatment groups did not differ significantly in OS, CSS, and DFS (*p* = 0.2681, 0.7208, and 0.3616, respectively). In multivariable Cox regression models, bladder preservation therapy remained non‐inferior to radical cystectomy in OS (adjusted hazard ratio [aHR] 1.08; 95% confidence interval [CI], 0.77–1.50; *p* = 0.6689), CSS (aHR, 1.06; 95% CI, 0.72–1.57; *p* = 0.7728), and DFS (aHR, 0.76; 95% CI, 0.46–1.27; *p* = 0.2929). Additionally, among patients ≥80 years, the use of bladder preservation therapy compared with radical cystectomy resulted in an equivalent OS, CSS and DSS.

**Conclusion:**

In Asian populations, bladder preservation therapy yielded similar survival outcomes as radical cystectomy in MIBC patients. Based on the results, it is evident that a multidisciplinary approach and shared decision‐making are recommended for bladder cancer treatment.

## 1 BACKGROUND

Bladder cancer is the tenth most common malignancy in the world, with 573,000 new cases and 213,000 deaths occurring in 2020 according to the estimation of the World Health Organization.^1^ The most common histology, urothelial carcinoma, accounts for 90%–95% of bladder cancer.^2^ Clinically, bladder cancer can be divided into three categories: non‐muscle‐invasive bladder cancer, muscle‐invasive bladder cancer (MIBC), and metastatic disease. MIBC accounts for 25%–30% of all bladder cancers.^3^

The standard treatment of MIBC is neoadjuvant chemotherapy followed by radical cystectomy. However, removal of the bladder increases the risk of perioperative complications and reduces the patient's quality of life. Given that the incidence of bladder cancer is higher in older adults, only a minority of MIBC patients older than 70 undergo radical cystectomy.^4^ Bladder preservation therapy is an alternative option to overcome these limitations of radical cystectomy for MIBC patients. A trimodality treatment strategy explored in the early 1990s included maximal transurethral resection of the bladder tumor (TUR‐BT) followed by concurrent chemoradiotherapy (CCRT).^5^, ^6^ The BC2001 randomized trial demonstrated that CCRT significantly improved locoregional control of MIBC, as compared to radiotherapy alone.^7^ Subsequently, several studies showed that trimodality bladder preservation therapy has a favorable survival outcome for MIBC patients, with a 5‐year overall survival (OS) rate of ~50%–60%.^8^, ^9^ The National Comprehensive Cancer Network (NCCN) guideline lists both bladder preservation therapy and radical cystectomy as main treatment strategies.

Major improvements in bladder preservation therapy have made it a more tolerable and acceptable treatment option for MIBC patients. However, no prospective randomized studies have compared the efficacy of bladder preservation therapy to that of radical cystectomy. The purpose of this study is to use propensity score matched analysis to compare the survival outcomes of MIBC patients treated with bladder preservation therapy versus radical cystectomy and to analyze the potential predictive factors.

## 2 METHODS

### 2.1 Study design

In this retrospective cohort study, using the Taiwan Cancer Registry (TCR) database and the Taiwan National Health Insurance Research Database (NHIRD), we identified MIBC patients treated with bladder preservation therapy or radical cystectomy between January 1, 2008 and December 31, 2018. This study was approved by the Chi‐Mei Medical Center Institutional Review Board (IRB; CMFHR11205010). The IRB waived the need for individual informed consent because the TCR database includes no personally identifiable information and all data were analyzed anonymously. The protocol was performed in accordance with relevant guidelines.

The tumor locations and histologic subtypes in the TCR database were recorded using the coding of *International Classification of Disease for Oncology, 3rd Edition*. We identified the urinary bladder for the tumor location as C67.0 through C67.9 and transitional cell carcinoma for the histologic type as 8120. Patients with non‐urothelial histology, clinical T1 or T4b classification, distant metastasis, or incomplete data were excluded. Demographic and clinical data included age, gender, clinical T classification, clinical N classification, Charlson Comorbidity Index (CCI) score, and comorbidities. The diagnostic codes of comorbidities in the NHIRD were recorded using the *International Classification of Diseases, 9th Revision* before 2015 and the *International Classification of Diseases, 10th Revision* thereafter. Parameters used for propensity score matching were the baseline assessment and the impact of tumor stage on patients, including age, gender, clinical stage, cT classification, and CCI score. One patient with bladder preservation therapy was matched to two patients receiving radical cystectomy. The bladder preservation therapy group was defined as those receiving a curative radiation dose of 60–66 Gray (gy) and chemotherapy within 28 days of radiation. The events of interest were the time to OS and disease‐free survival (DFS) during the follow‐up period. In addition, to detect the reduction in OS, cancer‐specific survival (CSS) patients who died from not MIBC were considered to be censored, was also analyzed. The maximum follow‐up duration of the study subjects was 5 years, and those who withdrew or were lost to follow‐up were right‐censored on December 31, 2018.

### 2.2 Statistical analysis

The patient distribution prior to and after propensity score matching between bladder preservation therapy and radical cystectomy was estimated using Pearson's chi‐square test for categorical variables and the Wilcoxon ranked sum test for continuous variables. A nearest‐neighbor greedy matching algorithm was used to enable the propensity score matching function. Following the matching process, the balanced distribution of the baseline information and tumor stage between the two groups was evaluated using absolute standardized mean differences (SMD), with a determined threshold of 0.2. The trends of OS, CSS, and DFS between groups were illustrated using the Kaplan–Meier method and the differences were compared using the log‐rank test. Risk was presented as hazard ratios (HRs) with 95% confidence intervals (95% CIs) and calculated using the Cox proportional hazard model after adjusting for other confounding variables. Statistically significance was set at *p* < 0.05. Multivariable Cox regression models were conducted to estimate the effect of comorbidities and smoking on OS, CSS, and DFS. Additionally, stratified analyses for MIBC patients who were older than 80 years old were used to reveal the interaction between the events and age. All statistical analyses were performed using SAS 9.4 for Windows (SAS Institute, Inc., Cary, NC, USA). Kaplan–Meier curves were plotted using STATA version 12 (Stata Corp., College Station, TX, USA).

## 3 RESULTS

### 3.1 Patient characteristics

**FIGURE 1:**
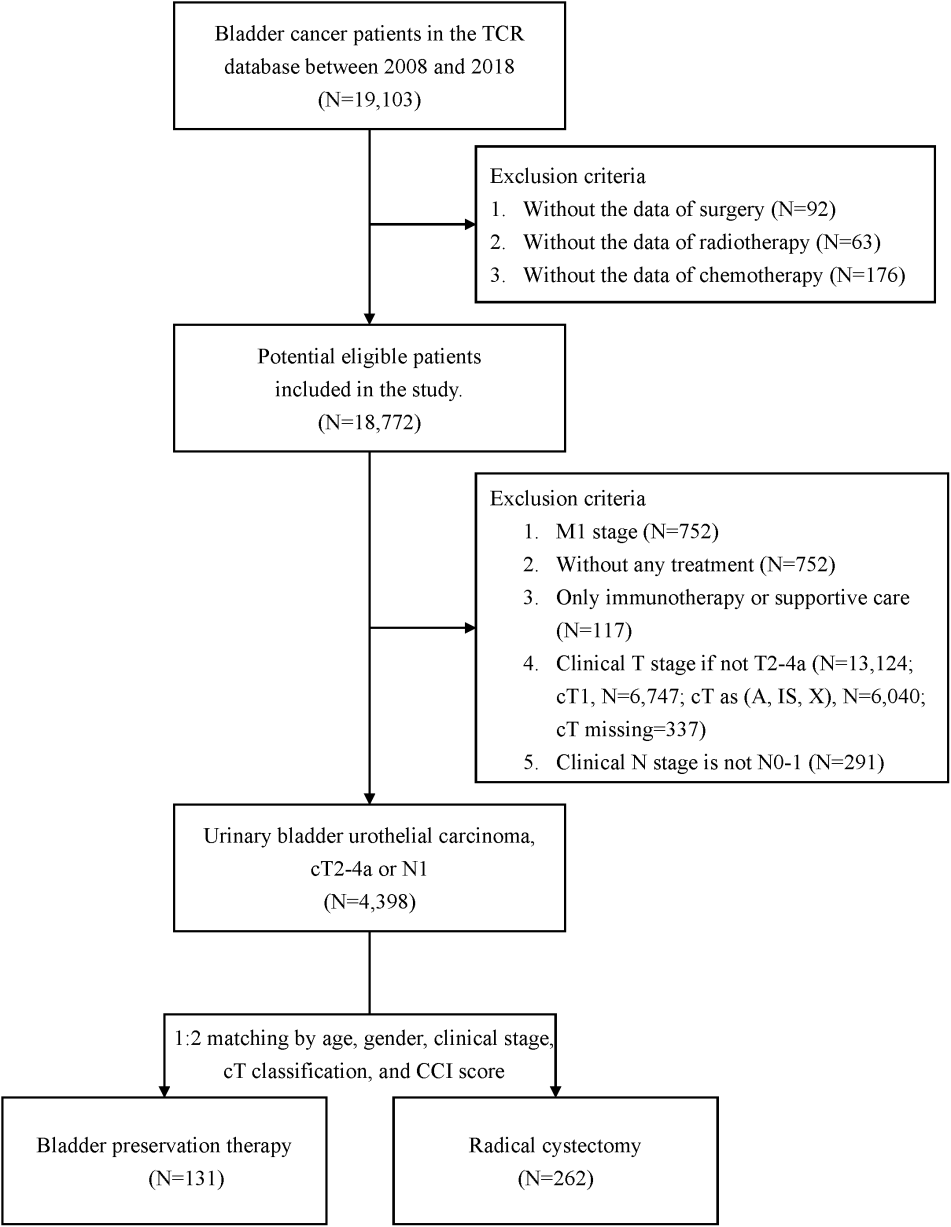
Flow chart of subject selection.

The flowchart of patient selection for this study is summarized in Figure 1. Overall, 19,103 patients with bladder cancer were extracted from the TCR database. Of the 4398 MIBC patients who met the inclusion criteria, those who underwent bladder preservation therapy were significantly older than those receiving radical cystectomy (*p* = 0.001) **(**Table S1 **)**. After propensity score matching, we analyzed 131 (33.3%) patients who underwent bladder preservation therapy and 262 (66.7%) patients who underwent radical cystectomy. These two treatment groups were comparable in terms of age, gender, clinical stage, cT classification, and CCI score with the SMD less than 0.2 (Table S2).

The demographic characteristics of the patients after propensity score matching are displayed in Table 1. The median age was 77. There were 274 (69.72%) males and 119 (30.28%) females. Clinical T classification was 151 (38.42%) in T2, 121 (30.79%) in T3, and 121 (30.79%) in T4a. Clinical N classification was 340 (86.51%) in N0 and 53 (13.49%) in N1. The chemotherapy regimens are summarized in Table S3. The most commonly used agents for CCRT were cisplatin, followed by carboplatin. There were still some MIBC patients who received self‐paid anticancer agents such as immunotherapy. The self‐paid drugs were not recorded in the NHIRD and TCR database.

**TABLE 1:** Baseline characteristics of MIBC patients treated with bladder preservation therapy or radical cystectomy after propensity score matching, *n* = 393.

	Total (*N* = 393)	Bladder preservation therapy (*N* = 131)	Radical cystectomy (*N* = 262)	*p*‐value
Age (years), median (Q1–Q3)	77 (70–84)	77 (70–83)	77 (70–84)	0.9002
Age group (years)				
<80	234	78 (59.54)	156 (59.54)	1.0000
≧80	159	53 (40.46)	106 (40.46)	
Gender				
Male	274	91 (69.47)	183 (69.85)	0.9381
Female	119	40 (30.53)	79 (30.15)	
cT classification				
2	151	50 (38.17)	101 (38.55)	0.9881
3	121	41 (31.30)	80 (30.53)	
4a	121	40 (30.53)	81 (30.92)	
cN classification				
0	340	115 (87.79)	225 (85.88)	0.6016
1	53	16 (12.21)	37 (14.12)	
Clinical stage				
2	148	49 (37.40)	99 (37.79)	0.8347
3	155	54 (41.22)	101 (38.55)	
4	90	28 (21.37)	62 (23.66)	
CCI score, mean ± SD	1.86 ± 2.07	1.96 ± 2.12	1.80 ± 2.05	0.4696
CCI				
0	140	44 (33.59)	96 (36.64)	0.5403
1–2	141	45 (34.35)	96 (36.64)	
≧3	112	42 (32.06)	70 (26.72)	
Comorbidities				
DM	91	34 (25.95)	57 (21.76)	0.3523
HTN	200	65 (49.62)	135 (51.53)	0.7213
CKD	41	12 (9.16)	29 (11.07)	0.5596
COPD	29	15 (11.45)	14 (5.34)	0.0290
Smoking status				
Non‐smoker	210	64 (48.85)	146 (55.73)	0.0406
Smoker (current/quit)	90	26 (19.85)	64 (24.43)	
Missing	93	41 (31.30)	52 (19.85)	
Time to follow‐up, median (Q1–Q3)	1.30 (0.57–2.92)	1.20 (0.59–3.35)	1.37 (0.56–2.85)	0.7776
Time to death within 5 years, median (Q1–Q3)	0.94 (0.50–1.76)	0.92 (0.57–1.73)	0.94 (0.49–1.85)	0.8885
Death within 5 years	248	92 (70.23)	156 (59.54)	0.0385
Recurrence or death within 5 years, total n = 158	82	22 (53.66)	60 (51.28)	0.7933
Time to recurrence or death within 5 years, median(Q1‐Q3)	0.96 (0.59–1.85)	1.59 (0.80–3.56)	0.83 (0.56–1.59)	0.0154

Abbreviations: CCI, Charlson Comorbidity Index; CKD, chronic kidney disease; COPD, chronic obstructive pulmonary disease; DM, diabetes mellitus; HTN, hypertension; MIBC, muscle‐invasive bladder cancer; SD, standard deviation.

*Note*: *p*‐value was calculated from Pearson's Chi‐square for categorical variables. Wilcoxon rank sum test was used to comparing the medians between the two groups.

### 3.2 Survival outcomes

**FIGURE 2:**
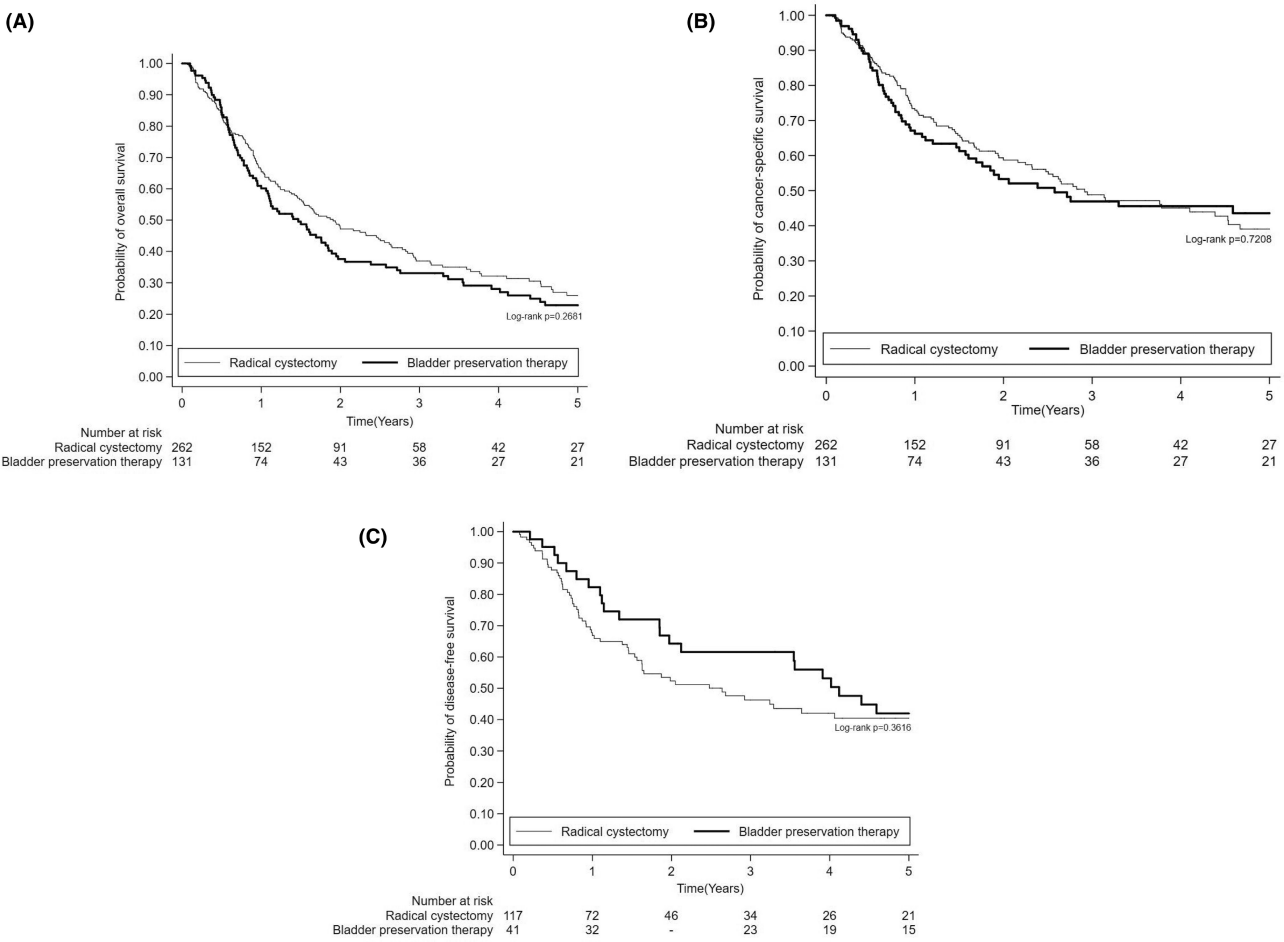
Kaplan–Meier plots of (A) overall survival, (B) cancer‐specific survival, and (C) disease‐free survival of muscle‐invasive bladder cancer patients receiving bladder preservation therapy versus radical cystectomy after propensity score matching.

During the 5‐year follow‐up period, the median duration was 15.6 months. There were no statistically significant differences between bladder preservation therapy and radical cystectomy in OS (*p* = 0.2681), CSS (*p* = 0.7208), and DFS (*p* = 0.3616) (Figure 2). After controlling for the variables, bladder preservation therapy remained non‐inferior to radical cystectomy in OS (adjusted hazard ratio [aHR], 1.08; 95% CI, 0.77–1.50; *p* = 0.6689), CSS (aHR, 1.06; 95% CI, 0.72–1.57; *p* = 0.7728), and DFS (aHR, 0.76; 95% CI, 0.46–1.27; *p* = 0.2929) **(**Tables 2 and 3 **)**. Additionally, among patients ≥80 years, the use of bladder preservation therapy compared with radical cystectomy resulted in an equivalent OS (aHR, 1.08; 95% CI, 0.73–1.60; *p* = 0.7079), CSS (aHR, 1.04; 95% CI, 0.65–1.67, *p* = 0.8790), and DFS (aHR, 0.75; 95% CI, 0.28–2.04; *p* = 0.5704) (Table 4).

**TABLE 2:** Adjusted HRs for 5‐year overall survival and cancer‐specific survival of MIBC patients receiving bladder preservation therapy versus radical cystectomy, *n* = 393.

	Total	Death	Adjusted HR (95% CI)	*p*‐value	Death caused by cancer	Adjusted HR (95% CI)	*p*‐value
Treatment							
Bladder preservation therapy	131	92 (70.23)	1.08 (0.77–1.50)	0.6689	60 (45.80)	1.06 (0.72–1.57)	0.7728
Radical cystectomy	262	156 (59.54)	Ref.		112 (42.75)	Ref.	
Comorbidity							
DM	91	61 (67.03)	0.85 (0.51–1.44)	0.5506	39 (42.86)	0.69 (0.35–1.38)	0.2980
HTN	200	136 (68.00)	0.96 (0.66–1.40)	0.8253	93 (46.5)	0.93 (0.59–1.46)	0.7501
CKD	41	31 (75.61)	1.69 (0.87–3.31)	0.1241	19 (46.34)	1.54 (0.68–3.48)	0.3024
COPD	29	22 (75.86)	1.04 (0.54–2.01)	0.8982	13 (44.83)	0.87 (0.39–1.94)	0.7285
Smoking status							
Non‐smoker	210	129 (61.43)	Ref.		93 (44.29)	Ref.	
Smoker (current/quit)	90	50 (55.56)	1.08 (0.65–1.81)	0.7591	37 (41.11)	0.94 (0.51–1.73)	0.8445
Missing	88	63 (71.59)	1.17 (0.77–1.77)	0.4627	42 (45.16)	0.63 (0.37–1.06)	0.0835

Abbreviations: CI, confidence interval; CKD, chronic kidney disease; COPD, chronic obstructive pulmonary disease; DM, diabetes mellitus; HR, hazard ratio; HTN, hypertension; MIBC, muscle‐invasive bladder cancer.

**TABLE 3:** Adjusted HRs for 5‐year disease‐free survival of MIBC patients receiving bladder preservation therapy versus radical cystectomy, *n* = 158.

	Total	Recurrence or death	Adjusted HR (95% CI)	*p*‐value
Treatment				
Bladder preservation therapy	41	22 (53.66)	0.76 (0.46–1.27)	0.2929
Radical cystectomy	117	60 (51.28)	Ref.	
Comorbidity				
DM	38	23 (60.53)	1.23 (0.64–2.37)	0.5402
HTN	76	46 (60.53)	1.40 (0.86–2.30)	0.1777
CKD	15	8 (53.33)	1.07 (0.48–2.42)	0.8646
COPD	10	8 (80.00)	1.22 (0.53–2.81)	0.6457
Smoking status				
Non‐smoker	78	36 (46.15)	Ref.	
Smoker (current/quit)	35	20 (57.14)	1.09 (0.58–2.03)	0.7963
Missing	45	26 (57.78)	1.07 (0.62–1.87)	0.8007

Abbreviations: CI, confidence interval; CKD, chronic kidney disease; COPD, chronic obstructive pulmonary disease; DM, diabetes mellitus; HR, hazard ratio; HTN, hypertension; MIBC, muscle‐invasive bladder cancer.

**TABLE 4:** Adjusted HRs for overall survival, cancer‐specific survival, and disease‐free survival in MIBC patients ≥80 years receiving bladder preservation therapy versus radical cystectomy.

	OS, *n* = 159		CSS, *n* = 159		DFS, *n* = 41	
	Adjusted HR (95% CI)	*p*‐value	Adjusted HR (95% CI)	*p*‐value	Adjusted HR (95% CI)	*p*‐value
Overall						
Bladder preservation therapy	1.08 (0.73–1.60)	0.7079	1.04 (0.65–1.67)	0.8790	0.75 (0.28–2.04)	0.5704
Radical cystectomy	Ref.		Ref.		Ref.	
Comorbidity						
DM	0.69 (0.42–1.13)	0.1414	0.57 (0.31–1.05)	0.0713	1.00 (0.32–3.12)	0.9948
HTN	1.47 (0.97–2.22)	0.0692	1.41 (0.87–2.29)	0.1619	2.16 (0.82–5.73)	0.1209
CKD	1.55 (0.89–2.69)	0.1211	1.24 (0.60–2.53)	0.5607	0.87 (0.22–3.47)	0.8480
COPD	0.85 (0.43–1.70)	0.6494	0.65 (0.25–1.68)	0.3757	1.24 (0.35–4.38)	0.7335
Smoking status						
Non‐smoker	Ref.		Ref.		Ref.	
Smoker (current/quit)	0.82 (0.46–1.47)	0.5010	0.64 (0.31–1.33)	0.2330	0.90 (0.23–3.55)	0.8781
Missing	0.84 (0.53–1.33)	0.4562	0.78 (0.45–1.36)	0.3860	0.97 (0.36–2.67)	0.9580

Abbreviations: CI, confidence interval; CKD, chronic kidney disease; COPD: chronic obstructive pulmonary disease; DM, diabetes mellitus; HR, hazard ratio; HTN, hypertension; MIBC, muscle‐invasive bladder cancer.

## 4 DISCUSSION

This study, using an Asian nationwide cancer registry database, found no significant differences in 5‐year OS, CSS, and DFS between bladder preservation therapy and radical cystectomy among MIBC patients. The NCCN guideline currently recommends both bladder preservation therapy and radical cystectomy for MIBC patients. Certain studies have demonstrated that bladder preservation therapy appears to be effective and results in excellent local control rates in MIBC patients. However, no prospective randomized studies have compared bladder preservation therapy to radical cystectomy in MIBC patients. Additionally, most of the data in previous retrospective studies were collected from the US National Cancer Database (NCDB) and the Surveillance, Epidemiology and End Results (SEER), which have limited Asian representation. We utilized the TCR database with a relatively large Asian population to compare the efficacy of bladder preservation therapy and radical cystectomy in MIBC patients. The TCR database, established in 1979, is an informative nationwide database covering approximately 97% of all the cancer cases in Taiwan. The accuracy of diagnosis and treatment coding is periodically reviewed and verified.^10^ Additionally, we combined the data with that of the NHIRD to explore the impact of comorbid conditions on survival outcomes. This process allowed sufficient variables and complete treatment records for this study of a national Asian population.

Most retrospective studies have identified the benefit of bladder preservation therapy with the use of CCRT after TUR‐BT in MIBC patients. In a single‐arm retrospective study, Byun et al. reported a complete response rate of 60% and a partial response rate of 26% for bladder preservation therapy.^11^ A pooled analysis of long‐term outcomes from the Radiation Therapy Oncology Group (RTOG) trials showed a 5‐year OS of 57% and 5‐year disease‐specific survival of 71% in MIBC patients after bladder preservation therapy.^8^ This discrepancy may be due to the difference in patient selection. The Byun et al. study indicated a median age of 71.5 years for their patients. In contrast, the RTOG group had a median patient age of 66 years, with an exclusion of N1 patients. Following propensity score matching in our study, the median age rose to 77 years, and we specifically included N1 patients in our cohort. Consequently, our findings revealed a less favorable 5‐year overall survival (OS), as elucidated in the revised manuscript. However, some retrospective studies had conflicting results. Ritch et al. reported short‐term survival benefit of CCRT in the first year, but higher mortality risk after 2 years.^12^ Fully 30%–40% of their patients had non‐urothelial histologic types of diseases. Those with bladder cancer with adenocarcinoma or squamous cell carcinoma had worse survival outcomes associated with bladder preservation therapy compared to cystectomy.^13^ Lin et al. analyzed the NCDB and showed that patients treated with cystectomy had better OS compared to those receiving CCRT before propensity score matching, whereas OS was similar between groups after propensity score matching.^14^ In a retrospective multi‐institutional study, Zlotta et al. found no differences in CSS and DFS between radical cystectomy and bladder preservation therapy.^15^ In concordance with larger‐scale population‐based studies with propensity score matching, our study demonstrated similar survival outcomes for bladder preservation therapy and radical cystectomy in MIBC patients in univariable and multivariable analyses. Additionally, we also enrolled some patients with clinical N1 classification disease. According to the current NCCN guideline, bladder preservation therapy is recommended for T2‐4a or N1 patients at the category 1 evidence level. This data emphasizes that multidisciplinary consultation with various specialists leads to better decision‐making and optimal care in MIBC patients. Studies in other types of cancer support improved outcomes in patients who receive treatment via the multidisciplinary approach.^16^

In our patients ≥80 years, bladder preservation therapy resulted in similar OS, CSS, and DFS compared with radical cystectomy. Similar retrospective observational studies have conflicting results. Seisen et al. conducted a retrospective analysis from the NCDB to identify similar survival outcomes for bladder preservation therapy and radical cystectomy in older individuals because of the perioperative mortality.^17^ Patients aged 75 years and younger had similar post‐treatment mortality after bladder preservation therapy or radical cystectomy, whereas older patients risked greater post‐treatment mortality after radical cystectomy.^18^ In contrast, Williams et al. analyzed the SEER database to find that patients aged more than 65 years receiving bladder preservation therapy had worse OS and CSS than those receiving radical cystectomy.^19^ Notably, the total median number of fractions delivered was 18 in the SEER study, where radiotherapy fraction data was available for half of the patients receiving bladder preservation therapy. A pooled analysis of the RTOG trials showed that patients aged more than 75 years had similar completion rates of radiotherapy >60 Gray (Gy) as younger patients, indicating that older patients tolerated definitive dosages of radiation.^8^

We also investigated predictive factors for survival outcomes by treatment in MIBC patients. We applied univariable and multivariable analyses to estimate the impact of comorbid conditions, including diabetes, hypertension, chronic kidney disease (CKD), and chronic obstructive pulmonary disease (COPD), factors seldom analyzed in previous studies. None of the comorbid conditions were significantly correlated with OS, CSS, and DFS in this study. The overall and stratified results indicated that the choice of treatment did not significantly impact patient survival outcomes. Some studies have identified predictive factors of decreased survival outcomes, such as age, advanced tumor stage, poor baseline performance status, and presence of tumor‐associated carcinoma in situ.^12^, ^20^, ^21^ Hamano et al. demonstrated that MIBC patients with preoperative CKD had worse survival outcomes than those without preoperative CKD.^22^ Zhang et al. identified that smoking‐related COPD was associated with worse overall survival than that of nonsmokers without COPD, in terms of both bladder cancer and all‐cause mortality, in MIBC patients receiving bladder preservation therapy.^23^ The European Association of Urology guideline emphasizes the importance of patient selection. However, an instructional consensus is lacking on how to make decisions that best suit the patients. Between bladder preservation therapy and radical cystectomy, the better choice for MIBC patients remains unknown based on current evidence. It will be important to identify the individuals who would be most benefit from each type of treatment. These findings could help guide treatment decisions.

Most of the patients in this study who received CCRT were treated with cisplatin‐based regimens. To date, cisplatin is the most widely used radiosensitizer and chemotherapeutic agent against urothelial carcinoma. Kumar et al. showed that bladder preservation therapy using preferred chemotherapy regimen, such as cisplatin, had similar outcomes to cystectomy. However, bladder preservation therapy with a non‐preferred chemotherapy regimen was associated with poorer survival.^24^ Some chemotherapeutic agents are also effective against urothelial carcinoma, such as carboplatin, gemcitabine, fluorouracil, and mitomycin C. Kobayashi et al. conducted a retrospective study of 35 MIBC patients and suggested radiation with concurrent gemcitabine and cisplatin was effective for local control, with a 91% complete response rate.^25^ The addition of fluorouracil and mitomycin C to radiotherapy improved survival in the BC2001 trial.^7^ CCRT using mitomycin C and capecitabine was efficient and well tolerated in a retrospective study of 71 MIBC patients.^26^ However, these regimens were rarely used for bladder preservation therapy in this study; this difference may be due to physician and patient preference.

An ongoing randomized phase III trial, KEYNOTE 992 (Clinical Trial Registry: NCT04241185) is comparing the efficacy of bladder preservation therapy with CCRT plus pembrolizumab versus CCRT plus placebo in patients with previously untreated MIBC. Pembrolizumab, a programmed cell death protein 1 (PD‐1) immune checkpoint inhibitor, is a treatment option for patients with previously treated metastatic urothelial carcinoma, according to the randomized phase III KEYNOTE 045 trial.^27^ Recent advances in immunotherapy have demonstrated clinical activity of PD‐1 or programmed cell death ligand 1 (PD‐L1) immune checkpoint inhibitors in urothelial carcinoma.^28^, ^29^ Further studies are warranted to define the indication of immunotherapy at different stages of urinary bladder urothelial carcinoma, including metastatic bladder cancer, MIBC, and non‐MIBC. Combining bladder preservation therapy with immunotherapy may represent a novel way to improve outcomes in MIBC patients. Additional studies of novel agents may improve the treatment outcomes for MIBC patients who receive bladder preservation therapy.

This study has certain limitations. First, given that radical cystectomy is mainly suggested for medically fit patients, selection bias in treatment may exist. Additionally, the number of patients who received radical cystectomy were much higher than the patients who received bladder preservation therapy. To overcome this limitation, we carefully balanced and matched age, gender, clinical stage, cT classification, and CCI score to create comparable cohorts of each treatment group. Second, we could not report long‐term survival outcomes owing to the limited follow‐up periods. The 5‐year and 10‐year OS of MIBC is approximately 50%–60% and 30%–40%, respectively.^30^ A longer follow‐up period is necessary. Third, some missing data, such as smoking status, made the evaluation of variables more complicated. Smoking status is associated with decreased response rates to cisplatin‐based chemotherapy regimens and higher mortality rate in bladder cancer.^31^ Fourth, it is important to note that the NHIRD and Taiwan Cancer Registry lack comprehensive information on self‐paid anticancer agents, treatment‐related toxicity, quality of life, chemotherapy dosage, and treatment duration. In our data analysis, it was observed that the utilization of self‐paid anticancer agents among patients with MIBC was minimal. This study could not present treatment‐related side effects and thus we were unable to report real world safety outcomes. Lastly, maximal TUR‐BT prior to CCRT constitutes a crucial aspect of bladder preservation therapy. However, as is similar to many cancer registry databases, this specific information was absent from our nationalized database. We operated under the assumption that our patients adhered to the treatment guidelines and thus, the estimated effect of bladder preservation therapy could be larger than we observed in the survival analysis. Future prospective studies are still necessary to validate our finding.

## 5 CONCLUSION

This study demonstrated that survival outcomes for bladder preservation therapy were comparable to those of radical cystectomy in MIBC patients in Asian populations. A multidisciplinary approach and shared decision‐making are highly recommended for bladder cancer treatment. Studies should evaluate the potential predictive factors or biomarkers to help physicians identify candidates who would most benefit from either bladder preservation therapy or radical cystectomy.

## AUTHOR CONTRIBUTIONS

**Nai‐Wen Kang:** Conceptualization (lead); investigation (lead); methodology (lead); writing – original draft (lead). **Yin‐Hsun Feng:** Conceptualization (equal); project administration (lead). **Kuei‐Li Lin:** Conceptualization (equal); methodology (equal). **Yi‐Chen Chen:** Formal analysis (equal); visualization (lead). **Chung‐Han Ho:** Formal analysis (equal); supervision (equal); validation (lead). **Ching‐Chieh Yang:** Conceptualization (equal); supervision (lead); writing – review and editing (lead).

## FUNDING INFORMATION

Not applicable.

## CONFLICT OF INTEREST STATEMENT

The authors declared that they have no competing interests.

## ETHICS STATEMENT

This study was approved by the Chi‐Mei Medical Center Institutional Review Board (IRB; CMFHR11205010).

## CLINICAL TRIAL REGISTRATION

Retrospectively registered.

## Supporting information


**Table S1.** Baseline characteristics of MIBC patients treated with bladder preservation therapy or radical cystectomy before propensity score matching.

**Table S2.** The balanced distribution of the baseline information and tumor stage between MIBC patients treated with bladder preservation therapy or radical cystectomy.

**Table S3.** Chemotherapy regimens of study subjects.

## Data Availability

The data presented in current study are available on request from the corresponding author.
