# Supplementary material for: Comparative survival analysis of bladder preservation therapy versus radical cystectomy in muscle‐invasive bladder cancer
Source: Cancer Med. 2024 Feb 6;13(2):e6972. doi: 10.1002/cam4.6972 (PMC10844988; doi:10.1002/cam4.6972)
Supplement: Supplementary file 1 — Table S1. Baseline characteristics of MIBC patients treated with bladder preservation therapy or radical cystectomy before propensity score matching. [file CAM4-13-e6972-s001.docx]

**Supplementary Table 1.** **Baseline characteristics of MIBC patients treated with bladder preservation therapy or radical cystectomy before propensity score matching.**

|  | **Total**  **(N=4,398)** | **Bladder**  **preservation**  **therapy**  **(N=131)** | **Radical**  **cystectomy**  **(N=4,267)** | **p-value** |
| --- | --- | --- | --- | --- |
| **Age (years), median(Q1-Q3)** | 73(62-80) | 77(70-83) | 72(62-80) | <0.0001 |
| **Age group (years)** |  |  |  |  |
| <80 | 3,176 | 78 (59.54) | 3,098 (72.60) | 0.0010 |
| ≧80 | 1,222 | 53 (40.46) | 1,169 (27.40) |  |
| **Gender** |  |  |  |  |
| Male | 3,118 | 91 (69.47) | 3,027 (70.94) | 0.7145 |
| Female | 1,280 | 40 (30.53) | 1,240 (29.06) |  |
| **cT classification** |  |  |  |  |
| 2 | 2,885 | 50 (38.17) | 2,835 (66.44) | <0.0001 |
| 3 | 1,083 | 41 (31.30) | 1,042 (24.42) |  |
| 4a | 430 | 40 (30.53) | 390 (9.14) |  |
| **cN classification** |  |  |  |  |
| 0 | 4,126 | 115 (87.79) | 4,011 (94.00) | 0.0036 |
| 1 | 272 | 16 (12.21) | 256 (6.00) |  |
| **Clinical stage, missing=13** |  |  |  |  |
| 1 | 1 | 0 (0) | 1 (0.02) | <0.0001 |
| 2 | 2,789 | 49 (37.40) | 2,740 (64.41) |  |
| 3 | 1,286 | 54 (41.22) | 1,232 (28.96) |  |
| 4 | 309 | 28 (21.37) | 281 (6.61) |  |
| **CCI score, mean±SD** | 1.85±2.10 | 1.96±2.12 | 1.85±2.10 | 0.5467 |
| **CCI** |  |  |  |  |
| 0 | 1,562 | 44 (33.59) | 1,518 (35.58) | 0.7101 |
| 1-2 | 1,567 | 45 (34.35) | 1,522 (35.67) |  |
| ≧3 | 1,269 | 42 (32.06) | 1,227 (28.76) |  |
| **Comorbidities** |  |  |  |  |
| DM | 1,018 | 34 (25.95) | 984 (23.06) | 0.4393 |
| HTN | 2,175 | 65 (49.62) | 2,110 (49.45) | 0.9696 |
| CKD | 546 | 12 (9.16) | 534 (12.51) | 0.2515 |
| COPD | 313 | 15 (11.45) | 298 (6.98) | 0.0502 |
| **Smoking status** |  |  |  |  |
| Non-smoker | 2,338 | 64 (48.85) | 2,274 (53.29) | 0.0022 |
| Smoker (current/quit) | 1,192 | 26 (19.85) | 1,166 (27.33) |  |
| Missing | 868 | 41 (31.30) | 827 (19.38) |  |
| **Time to follow up, median (Q1-Q3)** | 1.93 (0.82–4.26) | 1.20 (0.59–3.35) | 1.96 (0.83–4.29) | 0.0034 |
| **Time to death within 5 years, median (Q1-Q3)** | 1.09 (0.53–2.09) | 0.92 (0.57–1.73) | 1.10 (0.53–2.13) | 0.2785 |
| **Death within 5 years** | 1,937 | 92 (70.23) | 1,845 (43.24) | <0.0001 |
| **Recurrence or death within 5 years, missing=1,694** | 1,172 | 22 (53.66) | 1,150 (43.18) | 0.1792 |
| **Time to Recurrence or death within 5 years, median(Q1-Q3)** | 0.89(0.49-1.86) | 1.59(0.80-3.56) | 0.88(0.49-1.84) | 0.0.156 |

P-value was calculated from Pearson’s Chi-square for categorical variables.Wilcoxon rank sum test was used to comparing the medians between the two groups.

Abbreviations: MIBC, muscle-invasive bladder cancer; CCI, Charlson Comorbidity Index; SD, standard deviation; DM, diabetes mellitus; HTN, hypertension; CKD, chronic kidney disease; COPD: chronic obstructive pulmonary disease.
