# Supplementary material for: Comparative survival analysis of bladder preservation therapy versus radical cystectomy in muscle‐invasive bladder cancer
Source: Cancer Med. 2024 Feb 6;13(2):e6972. doi: 10.1002/cam4.6972 (PMC10844988; doi:10.1002/cam4.6972)
Supplement: Supplementary file 2 — Table S2. The balanced distribution of the baseline information and tumor stage between MIBC patients treated with bladder preservation therapy or radical cystectomy. [file CAM4-13-e6972-s002.docx]

**Supplementary Table 2.** **The balanced distribution of the baseline information and tumor stage between MIBC patients treated with bladder preservation therapy or radical cystectomy.**

|  | **Total**  **(N=393)** | **Bladder preservation therapy**  **(N=131)** | **Radical cystectomy**  **(N=262)** | **SMD** |
| --- | --- | --- | --- | --- |
| **Age group (years)** |  |  |  |  |
| <80 | 234 | 78(59.54) | 156(59.54) | <0.0001 |
| ≧80 | 159 | 53(40.46) | 106(40.46) |  |
| **Gender** |  |  |  |  |
| Male | 274 | 91(69.47) | 183(69.85) | 0.0083 |
| Female | 119 | 40(30.53) | 79(30.15) |  |
| **cT classification** |  |  |  |  |
| 2 | 151 | 50(38.17) | 101(38.55) | 0.0165 |
| 3 | 121 | 41(31.30) | 80(30.53) |  |
| 4a | 121 | 40(30.53) | 81(30.92) |  |
| **Clinical stage** |  |  |  |  |
| 2 | 148 | 49(37.40) | 99(37.79) | 0.0645 |
| 3 | 155 | 54(41.22) | 101(38.55) |  |
| 4 | 90 | 28(21.37) | 62(23.66) |  |
| **CCI** |  |  |  |  |
| 0 | 140 | 44(33.59) | 96(36.64) | 0.1179 |
| 1-2 | 141 | 45(34.35) | 96(36.64) |  |
| ≧3 | 112 | 42(32.06) | 70(26.72) |  |

SMD was absolute standardized mean difference for testing the balance of covariance between the two groups.
