# Supplementary material for: Comparative survival analysis of bladder preservation therapy versus radical cystectomy in muscle‐invasive bladder cancer
Source: Cancer Med. 2024 Feb 6;13(2):e6972. doi: 10.1002/cam4.6972 (PMC10844988; doi:10.1002/cam4.6972)
Supplement: Supplementary file 3 — Table S3. Chemotherapy regimens of study subjects. [file CAM4-13-e6972-s003.docx]

**Supplementary Table 3.** **Chemotherapy regimens of study subjects.**

| **Regimens, n (%)** | **Total**  **(N=393)** | **Bladder preservation**  **therapy**  **(N=131)** | **Radical cystectomy**  **(N=262)** |
| --- | --- | --- | --- |
| Cisplatin | 81(20.61) | 26 (19.85) | 55(20.99) |
| Carboplatin | 71(18.07) | 25 (19.08) | 46(17.56) |
| Gemcitabine | 121(30.79) | 38 (29.01) | 83(31.68) |
| Methotrexate | 0(0) | 0(0) | 0(0) |
| Vinblastine | 20(5.09) | 6 (4.58) | 14(5.34) |
| Doxorubicin | 34(8.65) | 10 (7.63) | 24(9.16) |
